# Three-Decade Analysis of Inflammatory Bowel Disease in Türkiye: A Multicenter Study (1993-2024)

**DOI:** 10.5152/tjg.2025.25063

**Published:** 2025-06-23

**Authors:** Muhammed Bahaddin Durak, Yasir Furkan Cagin, Ayhan Balkan, Yusuf Coskun, Tarkan Karakan, Yavuz Cagir, Yavuz Beyazit, Ali Atay, Ali Can Erdem, Guray Can, Engin Altinkaya, Hakan Dursun, Orhan Coskun, Mehmet Rasit Ayte, Mevlut Hamamci, Ilyas Tenlik, Cem Simsek, Abdurrahman Sahin, Huseyin Koseoglu, Mustafa Akar, Kenan Kosar, Bulent Albayrak, Eylem Karatay, Murat Kekilli, Mehmet Asil, Hilmi Ataseven, Mehmet Cindoruk, Ilhami Yuksel

**Affiliations:** 1 Department of Gastroenterology, Hacettepe University Faculty of Medicine, Ankara, Türkiye; 2 Department of Gastroentrology, Malatya Turgut Özal University Faculty of Medicine, Malatya, Türkiye; 3 Department of Gastroenterology, Gaziantep University Faculty of Medicine, Gaziantep, Türkiye; 4 Department of Gastroenterology, Etlik City Hospital, Ankara, Türkiye; 5 Department of Gastroenterology, Gazi University Faculty of Medicine, Ankara, Türkiye; 6 Department of Gastroenterology, Yenimahalle Training and Research Hospital, Ankara Yıldırım Beyazıt University, Ankara, Türkiye; 7 Department of Gastroenterology, Çanakkale Onsekiz Mart University Faculty of Medicine, Çanakkale, Türkiye; 8 Department of Gastroenterology, Ankara Bilkent City Hospital, Ankara, Türkiye; 9 Department of Gastroenterology, Necmettin Erbakan University Faculty of Medicine, Konya, Türkiye; 10 Department of Gastroenterology, Doruk Nilüfer Hospital, Bursa, Türkiye; 11 Department of Gastroenterology, Cumhuriyet University Faculty of Medicine, Sivas, Türkiye; 12 Department of Gastroenterology, Atatürk University Faculty of Medicine, Erzurum, Türkiye; 13 Department of Gastroenterology, Ankara Atatürk Sanatoryum Education and Research Hospital, Ankara, Türkiye; 14 Department of Gastroenterology, Tokat Gaziosmanpaşa University Faculty of Medicine, Tokat, Türkiye; 15 Department of Gastroenterology, Hitit University Faculty of Medicine, Çorum, Türkiye; 16 Department of Gastroenterology, University of Health Sciences, Bursa Yüksek İhtisas Training and Research Hospital, Bursa, Türkiye; 17 Department of Gastroenterology, Karabük Education and Research Hospital, Karabük, Türkiye; 18 Department of Gastroenterology, Atatürk Education and Research Hospital, Erzurum, Türkiye; 19 Department of Internal Medicine, İstinye University Faculty of Medicine, İstanbul, Türkiye; 20 Department of Gastroenterology, Ankara Yıldırım Beyazıt University Faculty of Medicine, Ankara, Türkiye

**Keywords:** Crohn’s disease, inflammatory bowel disease, ulcerative colitis

## Abstract

**Background/Aims:**

Inflammatory bowel diseases (IBDs) are increasingly prevalent and challenging globally. Data regarding IBD frequency and severity between Europe and Asia are needed. The aim was to investigate the trend of IBD in Türkiye during the last 3 decades.

**Materials and Methods:**

The study was conducted retrospectively at 14 centers in Türkiye between June 1993 and March 2024.

**Results:**

Over 30 years, 4308 patients, of whom 2507 (58.2%) had ulcerative colitis (UC) and 1717 (39.9%) had Crohn’s Disease (CD), were included in the study. The overall median age at the onset of IBD was 34.43 (25.28-45.64) years; the age at onset of IBD was younger in CD compared to UC (32.72 vs. 35.52 years respectively, *P* < .001). The peak age onset range was 28-32 years in CD, whereas 23-27 years in UC. Overall, 2526 (58.6%) patients were male. The most common location was left-sided colitis in UC patients (45.1%), followed by extensive colitis (31.2%), and proctitis (23.7%), while ileal involvement in CD patients (45.2%), afterward ileocolonic (409%), and colonic (13.5%). Both illnesses are becoming increasingly prevalent. The UC/CD ratio tends to decrease over time. During the study period, 1577 (36.6%) patients received biologic treatment. During the study period, 418 (24.3%) underwent resective surgery for CD and 88 (3.5%) total colectomy for UC; the major abdominal surgery has declined over time.

**Conclusion:**

The frequency and characteristic features of IBD in Türkiye appear to be between Europe and Asia. Over time, while the usage of biologic therapy and the rate of CD have increased, the frequency of surgery has decreased.

**Main Points**

- The current study revealed that the frequency of diagnosis of overall inflammatory bowel disease (IBD) and its subgroups has been on an upward trend over the years, with ulcerative colitis (UC) being more common than Crohn’s disease (CD), though the UC/CD ratio is decreasing over time in Türkiye.
- The peak age onset range was 23-27 years in CD, whereas 28-32 years in UC.
- The rate of major abdominal surgery was on a decreasing trend over eras for both diseases.
- The frequency and characteristic features of IBD in Türkiye appear to be between Europe and Asia. The peak age at diagnosis was found to be younger than the global peak age.

## Introduction

Inflammatory bowel disease (IBD) is characterized by chronic intestinal inflammation, complicated by strictures, fistulas, and colon adenocarcinoma, and symptoms that negatively affect the quality of life.^1^ The burden of the disease varies significantly by region, and prevalence in certain areas is not well established. Incidence is increasing, particularly among women and the elderly.^2^ Approximately 6.9 million individuals globally suffer from IBD, with an estimated 7 million cases in Europe and the Americas alone by 2030.^3^

In Western countries, the shift from an agricultural to an industrial lifestyle after World War II, along with the replacement of fiber with fast-food-style diets, has led to a rapid increase in disease burden in the 21st century. At the turn of the 21st century, the incidence of the disease in Western countries plateaued, while pediatric-onset IBD continued to rise.^4^^,^^5^ The highest incidence has been reported in Scandinavian countries.^6^ Reports suggest that the incidence of IBD doubled in Taiwan between 1998 and 2011,^7^ increased tenfold in South Korea over 20 years, and surged 20- to 50-fold in Japan between 1965 and 1991.^8^

While current studies on the disease burden in Southeast Asia, the Far East, Europe, North America, and Oceania are well established, data on the prevalence of IBD in the Middle East and West Asia are insufficient. Understanding the global burden, trends, and demographic characteristics can help design appropriate healthcare and diagnostic screening strategies tailored to various regions. Gradual global information collection on IBD will facilitate the establishment of practice guidelines specific to each region. To address the knowledge gap regarding the burden of IBD and its clinical features in the region bridging the East and West, the current study investigates the disease trend in Türkiye over the past thirty years.

## Materials and Methods

### Study Population and Data

The study was conducted at 20 centers across various regions and 13 cities in Türkiye. The centers included in the research were selected based on clinicians’ abilities to diagnose, treat, and follow up with patients, as well as to record data. Patients diagnosed with ulcerative colitis (UC) and Crohn’s Disease (CD) from June 1993 to March 2024 were part of this study. Patients under 18 years of age, with less than 6 months of follow-up, those misdiagnosed, and duplicate cases were excluded from the research. Demographic details, diagnoses and subtypes, laboratory variables at diagnosis, treatment details, surgical history, and clinical outcomes were recorded. Disease subtypes, age, and gender were noted across different years and specific periods. Each center contributed data to a common civil medical record system. Missing data were obtained by examining each hospital’s medical record system and the national electronic medical record system. Patients from whom sufficient data could not be gathered were excluded from the study. Subsequently, the data were exported to a database and prepared for statistical analysis. The methodologies adhered to the 1964 Helsinki Declaration, its subsequent revisions, the ethical guidelines of the institutional research committee, or equivalent standards. The institutional review board of Ankara Bilkent City Hospital approved this study on MAY 8, 2024, granting approval number TABED 1-24-204. The capable administrative departments provided ethics approval to all participating centers. Informed consent was not obtained from participants due to the retrospective nature of the study.

### Patient Management

The patients were categorized into 3 groups: the non-biologic era (1993-2007), the early period of biologics (2008-2015), and the common era of biologics (2016-2024) based on the introduction of biologic treatments for IBD in 2008 and their widespread use in Türkiye by 2016. Treatment options prior to the availability of biologic therapies included azathioprine, mesalazine compounds (oral or local), and corticosteroids (local or systemic). Surgery served as an alternative when medical treatments failed. While anti-TNF-α, also known as tumor necrosis factor-alpha antagonists, were the first biologic agents used, various biological agents (including anti-integrins, IL-13/IL-24 antagonists, and Janus kinase inhibitors) were subsequently employed as medical treatments for moderate to severe disease. Further subtyping analysis was performed using demographic data, including age, gender, disease prevalence and severity, treatment, and clinical outcomes, based on the selected period. To determine the peak age range for disease onset, analysis was conducted utilizing 5-year age intervals.

### Definitions

Inflammatory bowel disease’s “age at diagnosis” refers to the date of an established diagnosis, regardless of the disease’s phenotype. In contrast, “total disease duration” reflects the interval between the initial diagnosis and the last clinical visit. The UC/CD ratio indicates the proportion of individuals diagnosed with UC compared to those diagnosed with CD each year. The Montreal classification served as the basis for the diagnosis and subtyping.^9^ Until 2017, the CD location was established by colonoscopy in conjunction with 1 or a combination of computed tomography, MR enterography, and double-contrast barium radiography, while MR enterography and colonoscopy were used as of 2017. For patients with CD, resective surgery has been identified as small- or large-bowel resections. Total colectomy is used for UC patients. Disease behavior refers to behavior that occurs at the onset of diagnosis or during follow-up. The same was considered for perianal involvement.

### Statistical Analysis

Data analysis was performed using the IBM SPSS Statistics for Windows, version 25.0 (IBM SPSS Corp.; Armonk, NY, USA) and Microsoft Excel, 2013. To determine the normality of the distribution of continuous variables in the study, the Kolmogorov-Smirnov test was employed. Normally distributed continuous variables were given as mean ± SD and were compared by using the one-way ANOVA test. Non-normally distributed continuous variables were given as median (interquartile range) and were compared by using the Kruskal-Wallis test followed by Tamhane’s T2 post hoc test. The Mann-Whitney U test was applied for the analysis of continuous variables, while the chi-square test or Fisher’s Exact test was utilized for categorical variables. A *P*-value < .05 was considered significant.

## Results

**Figure 1.**
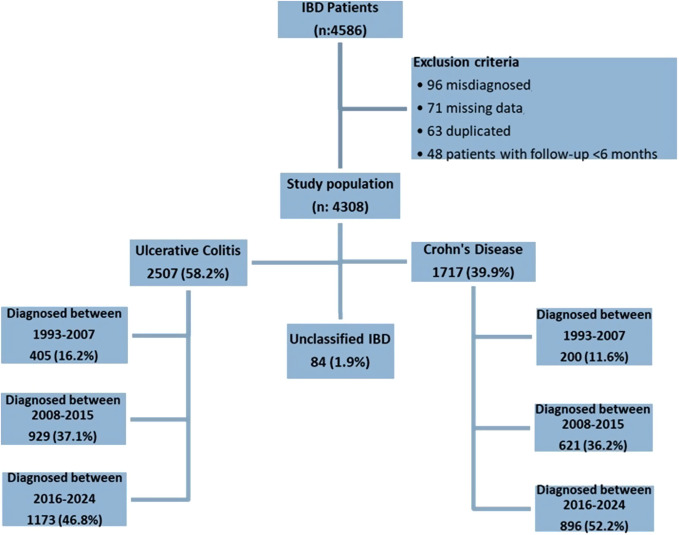
The inclusion criteria and study population.

A total of 4586 patients were followed during the study period. About 96 misdiagnosed, 71 missing data, 63 duplicated, and 48 patients with a follow-up period of <6 months were excluded from the study. The analysis includes the remaining 4308 patients. About 2507 (58.2%) patients had UC, 1717 (39.9%) had CD, and 84 (1.9%) had unclassified inflammatory bowel disease (UIBD). The inclusion criteria and study population are shown in a flowchart in Figure 1. In the overall study population, the median disease duration was 93.62 months (45.97-149.53), the median age at onset was 34.43 years (25.28-45.64), and 2526 (58.6%) patients were male. Age at onset of UC was older compared to CD [35.52 (25.79-47.85) vs. 32.72 (24.57-42.96) years, *P* < .001], while current smokers were more likely in CD [602 (35.1%) vs. 546 (21.8%), *P* < .001] (Table 1).

In CD and UC patients, the diagnosis was most frequently made during the common era of biologics [1173 (46.8%) vs 896 (52.2%), *P* < .001, respectively], and in U-IBD, it was made in the early period of biologics. Left-sided was the most frequent in UC (45.1%), ileal involvement was in CD (45.2%), and the inflammatory type of behavior was the most common (73.4%). The stricturing and penetrating diseases were 13.5% and 13%, respectively. Perianal involvement was present in 451 (26.3%) CD patients. Extraintestinal manifestations (EIMs) were 48.8%, 32.3%, and 24.1% in UIBD, CD, and UC, respectively, and were statistically significant for all 3 groups (*P* < .001). The most common EIMs in CD were peripheral arthralgia (16.5%), aphthous ulcer (8.7%), peripheral arthritis (5.5%), and ankylosing spondylitis (4.9%), respectively. Oral mesalazine was the most frequently administered conventional medical treatment for UC (79.7%) and UIBD (82.1%), while thiopurine was the most frequently prescribed therapy for CD (71.2%). 1577 patients (36.6%) received biological treatment during the study period. The most frequently used biological agent was infliximab (IFX) in the UC (14.2%) and CD (36.9%) subgroups and adalimumab (ADA) in the UIBD (6%) subgroup (Table 1).

Table 2 displays clinical, laboratory, and demographic information for UC patients based on the period of diagnosis. 405 (16.2%) UC patients were diagnosed in the non-biologic era (1993-2007), 929 (37.1%) in the early period of biologics (2008-2015), and 1173 (46.8%) in the common biologics era (2016-2024). The median age at onset of UC was 36.98 (27.79-44.97) in the non-biologic era, while 34.2 (24.62-48.51) for the common era of biologics (*P* < .034). Over the years, it was shown that UC onset occurred at younger ages. Initially, left-sided (50.9%), extensive colitis (37%), and proctitis (12.1%) were the most common locations; currently, left-sided (44%), extensive colitis (28.8%), and proctitis (27.2%) are the most common locations. The frequency order remained constant across all 3 periods, yet the trend of shifts in rates showed a statistically significant difference (*P* < .001). Peripheral arthralgia, ankylosing spondylitis, and aphthous ulcers showed a decreasing trend over the years (*P* < .001 for all parameters) in the overall and subgroup analysis of EIMs. There was no statistically significant difference in the other EIMs over the eras. The total colectomy rate was the highest in the non-biologic era subgroup (9.1%). It showed a decreasing trend in the early period of biologics (3.2%) and the common era of biologics (1.8%, (*P* < .001 for all periods) (Table 2).

Based on the diagnosis period, CD patients’ demographics, clinical, and laboratory data are shown in Table 3. Among CD patients, 200 (11.6%) were diagnosed in the non-biologic era, 621 (36.2%) in the early period of biologics, and 896 (52.2%) in the common era of biologics. Median age at onset of CD was younger in the non-biologic era group compared to the common era of biologics [29.08 (22.64-35.31) vs. 33.32 (24.25-44.19), *P* < .001]. In contrast to UC patients, the age at onset of CD tended to increase over the years. Crohn’s disease localization rates were ileocolonic (51.5%), ileal (33%), and colonic involvement (15.5%) in the non-biologic era, while in the common era of biologics, they were ileal (51.1%), ileocolonic (35.8%), and colonic (12.7%). There was a tendency for ileocolonic involvement to decline whereas ileal disease tended to rise (*P* < .001). Perianal involvement showed a tendency to decline (70 (35%) vs. 211 (23.5%), *P* = .003). As the disease’s behavior was analyzed by period, the rates of penetrating and stricturing diseases trended to decrease (*P* < .001 for all periods), while the rates of inflammatory behavior were trended to increase. The EIMs rate was comparable in each decade. Resective surgery was performed on 418 (24.3%) patients during the study period. Resective surgery rates by era were as follows: 58.5% during the non-biologic era, 26.9% during the early biologics period, and 15% during the common biologics era. The resective surgery rates were decreasing trend over time (*P* < .001 for all periods) (Table 3).

Inflammatory bowel disease diagnosis trend is displayed in Table 4. Both overall IBD and subgroup diagnoses tended to increase. The UC/CD ratio showed a decreasing trend from the non-biologic era to the common era of biologic. About 605 (14.3%) patients were diagnosed between 1993 and 2007, 405 (66.9%) had UC and 200 (33.1%) had CD, while of the 2069 patients diagnosed between 2016 and 2024, 1173 (56.7%) had UC, and 896 (43.3%) had CD. There was a growing tendency in CD even though more individuals had UC. In the last 2 years, it was determined that the rate of CD diagnosis exceeded that of UC (Table 5).

**Figure 2.**
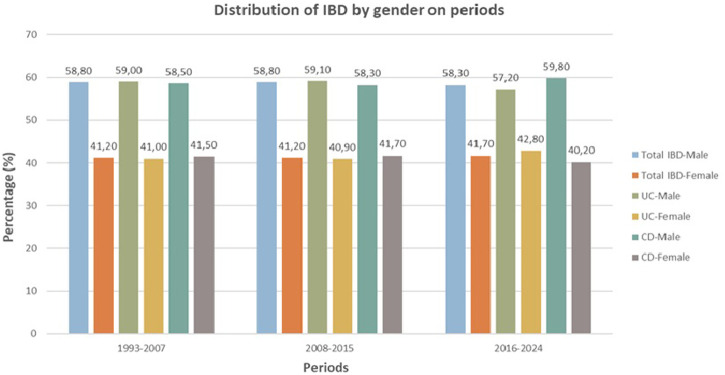
Distribution of IBD by gender on periods (CD, Crohn’s disease; IBD, Inflammatory bowel disease; UC, Ulcerative colitis).

**Figure 3.**
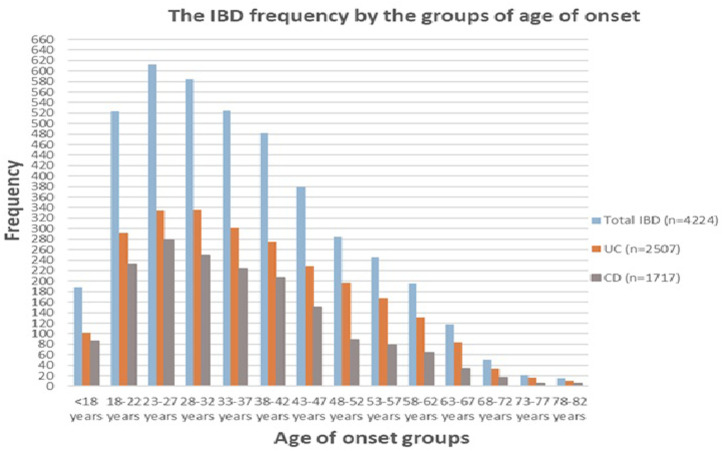
Inflammatory bowel disease diagnosis frequency based on 5-year age intervals (CD, Crohn’s disease; IBD, Inflammatory bowel disease; UC, Ulcerative colitis).

In UC patients, the median age at onset in the non-biologic era and Common period of biologics was (36.98 vs. 34.20 years, *P* < .001), and in CD patients, it was (29.08 vs. 33.32, *P* < .001), respectively. A decreasing trend in the median age at the onset of UC was observed over the eras. In contrast to UC patients, the age at onset of CD tended to increase over the years. Figure 2 shows the gender distribution graph by period. Male predominance was observed in both diseases. Gender distribution was comparable between the periods. The peak age onset range was 23-27 years in CD, whereas 28-32 years in UC (Figure 3).

The medical treatment trends for UC/CD patients by era are displayed in Tables 6 and 7. In UC, IFX was the most widely prescribed biological agent, and oral mesalazine was the most widely conventional treatment. In CD, IFX was the most widely prescribed biological agent, whereas thiopurine was the most widely conventional treatment. Conventional and biological treatment rates tended to rise over the eras.

## Discussion

The current study revealed that the frequency of diagnosing overall IBD and its subgroups has been on an upward trend over the years, with UC being more common than CD. However, Türkiye’s UC/CD ratio has decreased over time. Since 2022, the number of newly diagnosed CD patients has exceeded that of UC patients. The age of onset for CD has tended to increase over the years, in contrast to UC patients. The peak age of onset for CD was between 23 and 27 years, while it was between 28 and 32 years for UC. The most common manifestation of UC was left-sided, followed by extensive colitis and proctitis. It was determined that the rate of left-sided involvement tended to decrease, whereas the rate of proctitis tended to increase over time. Although ileocolonic involvement was detected more frequently in CD initially, it has decreased over time, while ileal involvement has tended to increase. Recently, the most frequent location for involvement was ileal, followed by ileocolonic and colonic involvement. Inflammatory behavior was detected most frequently, with a noted decrease in the trend of stricturing and penetrating disease, as well as perianal involvement. EIMs were detected more in CD than in UC across all periods. While EIMs tended to decrease in UC over time, they remained stable in CD. The rate of major abdominal surgery has shown a decreasing trend over eras for both diseases.

Over the past 50 years, IBD has become increasingly prevalent worldwide. The overall increase in life expectancy is contributing to the rising prevalence of older adults with IBD. Approximately 6.9 million individuals globally suffer from IBD, with an estimated 7 million cases in Europe and the Americas alone by 2030.^3^^,^^10^ The prevalence of IBD varies among populations and regions. While the disease’s incidence is higher in the West, it has been generally stable in Western countries over the last 20 years and has increased in Eastern countries.^11^ Given the rapid industrialization and socioeconomic changes happening in Asian countries, this growth is likely related to lifestyle and environmental factors.^12^ Following World War II, industrialization, rural-to-urban migration, and an increase in high-fat diets were linked to the rising incidence of IBD in Western countries.^13^ The rapid industrialization, increasing urban migration, and widespread consumption of a Western diet, coupled with genetic factors, may have contributed to the increase in the incidence of IBD in Türkiye in the 2000s.

In Western countries, the incidence of CD is higher, while UC is more prevalent in Eastern countries.^14^ In Western countries, the peak age of CD onset is observed in the 3rd-4th decades, with a second peak in the 6th-7th decades.^15^^,^ ^16^ In the East, the peak age of CD onset is similar to that in the West, and a recently reported second peak age range has been noted. A population-based retrospective study in the Asia-Pacific region reported that the second peak age range (40-44 years) is much younger than the second peak age in the West.^8^^,^ ^11^ The age at onset of UC among patients in Asia is slightly older or comparable to that among patients in the West (30-40 years in the West versus 35-44 years in Asia).^8^^,^ ^17^ The current study demonstrated that UC occurs more frequently than CD, with a declining UC/CD ratio over time (the UC/CD ratio was 2.03 in the non-biologic era compared to 1.31 in the biologic era). In this regard, it is comparable to Asia; however, in the past 2 years, the frequency of CD has exceeded that of UC, similar to trends in Europe. Another surprising finding in the current study was that the peak age of onset was between 23 and 27 years for CD, while it was between 28 and 32 years for UC. A considerably earlier peak age was observed compared to the East.

Studies in Western countries^18^ have revealed that the incidence of CD in women equals or exceeds that in men, while a male predominance has been reported in Eastern patients with CD.^11^^,^ ^19^ The age and gender distributions of patients with UC are almost similar between the East and the West.^20^^,^ ^21^ In the current study, the gender distribution in UC (58.2% male) was different from both the West and the East, although in CD (59.1% male), it was comparable to the East. Both diseases exhibit a male predominance in Türkiye.

In a comparable percentage of patients, CD has been documented to appear in the ileum, colon, and both the ileum and colon in the West.^17^^,^^22^ The most common involvement of CD, as shown by several research studies from the West, is isolated colonic disease; nevertheless, small bowel CD is also prevalent in Asia.^21^^-^^23^ Furthermore, the most common involvement of CD in Asia seems to be ileocolonic disease. Studies conducted in the West and Asia have demonstrated similar incidences of stricture, penetrating, and perianal disease in patients with CD.^24^^,^^25^ In this study, during the non-biologic era, ileocolonic location was the most common involvement in CD, similar to the East; in contrast, ileal involvement became the most common, akin to the West over the years.

As reported by Western population-based research, the percentage of cases with UC involvement that have proctitis is 30%-60%, left-sided colitis is 16%-40%, and extensive colitis is 18%-35%.^23^^,^^26^ They were reported as 25.0%-43.7%, 31.0%-31.4%, and 24.9%-39.0%, respectively, in Asian population-based studies. Comparing Asian data with population-based investigations, there has been a tendency for fewer cases of proctitis (8.5%-38.4%), more cases of left-sided colitis (29.7%-70.2%), and similar rates of extensive colitis (21.3%-42.4%).^21^^,^^27^^,^^28^ Proctitis and left-sided colitis were predominant in the East and West, respectively, while left-sided and extensive colitis were predominant in Türkiye.

According to some sources^25^^,^^29^^,^^30^ the prevalence of EIM in Asia is thought to be either somewhat lower or almost identical to that in the West (19%-25% vs. 21%-41%). Extraintestinal manifestation involvement sites differ in the East and West. In Asian individuals with UC, the joints are the most frequently affected site of EIM (2.0%-19.5%). Skin and ocular involvement come next, in that order.^24^ On the other hand, in Western countries, the most commonly related sites were the eye (iritis/uveitis in females; 3.8%) and primary sclerosing cholangitis (PSC) in males (3.0%). Compared to the West (1.6-7.0%), PSC linked to UC is less common in Asia (0.0%-1.7%).^31^ In Türkiye, the incidence of EIMs in IBD was comparable to the global average (27.9%). Extraintestinal manifestations were more prevalent in CD than in UC. Over time, the incidence of EIMs in both diseases has decreased. As in the East, the most common EIM site was the joints, followed by oral involvement. Major abdominal surgery has been declining over time, according to the current study. Surgery rates appear to be decreasing as the use of biological therapies becomes more widespread.

When previous studies on IBD prevalence in Türkiye were analyzed, Tozun et al’s^32^ 2009 study found that IBD prevalence was lower than in North and West Europe and closer to the East. The current study analyzed that IBD demographics in Türkiye approached those of North and West Europe over time. Both studies found similar male predominance in both diseases and more EIM in CD. Can et al^33^ showed that the prevalence of IBD tended to increase in Türkiye between 2004 and 2013, which is consistent with this study. Iltar et al^34^ found that the frequency of UC was higher and that there was a female predominance in UC, which contrasts with the current analysis. This may be related to the fact that Iltar et al^34^ included patients diagnosed in the Mediterranean region in their study. Tezel et al^35^ demonstrated that the first peak of IBD occurs between the ages of 10 and 30, while the second peak is between 50 and 70. In contrast to the wide age distribution presented by Tezel et al,^35^ the peak ages for CD were between 23 and 27 years, and 28 to 32 years for UC in the current study. Contrary to the previously discussed Turkish epidemiological studies, the present analysis demonstrated a declining trend in major abdominal surgery for IBD and a decreasing trend in the UC/CD ratio over time.

When studies analyzing the demographic characteristics of IBD in neighboring countries in the Middle East were conducted, Israeli-based research^36^ found that, similar to the current study, there was a tendency for the age of disease onset to decrease and for its prevalence to increase over time in CD patients. Al-Shamali et al^37^ reported that the prevalence of UC in the Kuwait cohort was on an increasing trend, with the peak age of diagnosis occurring in the third decade, as seen in the current analysis. In a 2019 multicentric analysis of IBD in the Middle East and North Africa,^38^ the highest rate was reported in Jordan, followed by Türkiye and Kuwait. The largest increase between 1990 and 2019 was recorded in Türkiye (50.7 per 100 000 in 1990, 98.9 per 100 000 in 2019). It was reported that the incidence and prevalence of IBD in the Middle East and North Africa increased from 1990 to 2019, with a higher rise in males and a decreasing trend in IBD-related mortality rates. The current analysis reflects the similarities in IBD demographics across the Middle East.

The study’s limitations included the inability to determine prevalence separately for each geographic region in the country. The centers included in the study follow up with patients both locally and from various provinces; therefore, geographic subgrouping regarding the prevalence of the disease could not be made. Since upper GI endoscopy is not routinely performed on CD patients, the frequency of CD with upper GI involvement could not be assessed. The strength of the study was that the relevant data were extracted from a common civil medical record system. As patient data were entered into the record system algorithmically, the data across all centers were standardized. The national medical record system, accessible to all clinicians, also contributed to this standardization. The subgroups were determined as references for the eras when biological treatments were first employed and widely utilized in Türkiye. Biological treatments enhanced the optimal design of the study as they influence disease course and clinical outcomes.

The frequency and characteristics of IBD in Türkiye initially ranged between Europe and Asia but have become increasingly similar to those in Europe over time. Notably, the peak age at diagnosis was younger than the global peak age. Over time, the use of biological therapy and the rate of CD increased significantly, while the frequency of surgery tended to decrease. Current real-world data analysis will aid in determining the global burden of the disease and its characteristics. Future national studies, featuring geographic subgroup analyses, will assist in clarifying regional prevalence in Türkiye.

## Figures and Tables

**Table 1.** Demographic, Clinical, and Laboratory Data of the Study Group and Subgroups According to Inflammatory Bowel Disease Type^a^

	**Study Group** (n = 4308)	**UC** (n = 2507, 58.2%)	**CD** (n = 1717, 39.9%)	**UIBD** (n = 84, 1.9%)	*P*
Age at diagnosis, years	34.43 (25.28-45.64)	35.52 (25.79-47.85)	32.72 (24.57-42.96)	36.15 (25.6-47.4)	<.001*
Smoking, n (%) None Ex Current	2303 (53.5) 835 (19.4) 1170 (27.2)	1456 (58.1) 505 (20.1) 546 (21.8%)	808 (47.1) 307 (17.9) 602 (35.1)	39 (46.4) 23 (27.4) 22 (26.2)	<.001*
Family history of IBD, n (%)	684 (15.9)	446 (17.8)	222 (12.9)	16 (19)	<.001*
BMI (kg/m^2^)	25.77 (23.26-28.65)	25.95 (23.53-28.72)	25.54 (22.89-28.55)	25.29 (22.99-28.81)	.008*
Period of diagnosis, n (%) 1993-2007 2008-2015 2016-2024	621 (14.4) 1586 (36.8) 2101 (48.8)	405 (16.2) 929 (37.1) 1173 (46.8)	200 (11.6) 621 (36.2) 896 (52.2)	16 (19) 36 (42.9) 32 (38.1)	<.001***
UC disease extension, n (%) Proctitis Left-sided Extensive	- - -	595 (23.7) 1131 (45.1) 781 (31.2)	- - -	- - -	-
CD disease location, n (%) Ileal Colonic Ileocolonic Upper GIS	- - - -	- - - -	776 (45.2) 232 (13.5) 702 (40.9) 7 (0.4)	- - - -	-
CD disease behavior, n (%) Inflammatory Stenosing Penetrating	- - -	- - -	1261 (73.4) 232 (13.5) 224 (13)	- - -	-
CD perianal disease, n (%) Total Isolated perianal disease	- -	- -	451 (26.3) 30 (1.7)	- -	-
Major abdominal surgery, n (%) Resection Total colectomy	421 (9.8) 89 (2.1)	- 88 (3.5)	418 (24.3) -	3 (3.6) 1 (1.2)	<.001**** <.001***
Conventional medications, n (%) Mesalazine oral Sulfasalazine Budesonide Steroids Thiopurine Methotrexate	3072 (71.3) 264 (6.1) 685 (15.9) 1858 (43.1) 2187 (50.8) 313 (7.3)	1999 (79.7) 108 (4.3) 102 (4.1) 1031 (41.1) 941 (37.5) 49 (2)	1004 (58.5) 148 (8.6) 581 (33.8) 798 (46.5) 1223 (71.2) 259 (15.1)	69 (82.1) 8 (9.5) 2 (2.4) 29 (34.5) 23 (27.4) 5 (6)	<.001*** <.001* <.001*** <.001* <.001*** <.001**

Significant *P* values are in bold.

BMI, body mass index; CD, Crohn’s disease; GIS, gastrointestinal system; IBD, inflammatory bowel disease; UC, ulcerative colitis; UIBD, unclassified inflammatory bowel disease.

*Statistically significant difference is between UC-CD subgroups.

**Statistically significant differences are between UC-CD and UC-UIBD subgroups.

***Statistically significant differences are between UC-CD and CD-UIBD subgroups.

****Statistically significant differences are between UC-CD, UC-UIBD, and CD-UIBD subgroups.

*****Statistically significant difference is between CD-UIBD subgroups.

^a^Results are expressed as median (interquartile range), or frequency (%).

**Table 2.** Demographic, Clinical, and Laboratory Data of the Subgroups According to the Period of Diagnosis in Patients with Ulcerative Colitis^a^

	Non-Biologic Era (<1993-2007) (n = 405, 16.2%)	Early Period of Biologics (2008-2015) (n = 929, 37.1%)	Common Era of Biologics (2016-2024) (n = 1173, 46.8%)	*P*
Age at diagnosis, years	36.98 (27.79-44.97)	36.44 (27.02-48.36)	34.2 (24.62-48.51)	.034*
UC disease extension, n (%) Proctitis Left-sided Extensive	49 (12.1) 206 (50.9) 150 (37)	227 (24.4) 409 (44) 293 (31.5)	319 (27.2) 516 (44) 338 (28.8)	<.001****
Extraintestinal manifestations, n (%) Peripheral arthralgia Ankylosing spondylitis Aphthous ulcer	116 (28.6) 50 (12.3) 17 (4.2) 36 (8.9)	247 (26.6) 82 (8.8) 23 (2.5) 35 (3.8)	241 (20.5) 79 (6.7) 20 (1.7) 43 (3.7)	<.001*** .002***** .018***** <.001****
Major abdominal surgery, n (%) Total colectomy	37 (9.1)	30 (3.2)	21 (1.8)	<.001****
Baseline endoscopic Mayo score	2 (2-3)	2 (2-3)	2 (2-3)	.485
Baseline partial Mayo score	6 (5-7)	6 (5-7)	7 (5-8)	.231

Significant *P* values are in bold.

UC, ulcerative colitis.

*No statistically significant difference was found between subgroups.

***Statistically significant differences are between NBE-CEB and EPB-CEB subgroups.

****Statistically significant differences are between NBE-CEB and NBE-EPB subgroups.

******Statistically significant differences are between EPB-CEB subgroups.

^a^Results are expressed as median (interquartile range) or frequency (%).

**Table 3.** Demographic, Clinical, and Laboratory Data of the Subgroups According to the Period of Diagnosis in Patients with Crohn’s Disease^a^

	**Non-Biologic Era** (<1993-2007) (n = 200, 11.6%)	**Early Period of Biologics** (2008-2015) (n = 621, 36.2%)	**Common Era of Biologics** (2016-2024) (n = 896, 52.2%)	*P*
Age at diagnosis, years	29.08 (22.64-35.31)	33.72 (25.49-43.67)	33.32 (24.25-44.19)	<.001*
CD disease location, n (%) Ileal Colonic Ileocolonic Upper GIS	66 (33) 31 (15.5) 103 (51.5) -	252 (40.6) 87 (14) 278 (44.8) 4 (0.6)	458 (51.1) 114 (12.7) 321 (35.8) 3 (0.3)	<.001***
CD disease behavior, n (%) Inflammatory Stenosing Penetrating	99 (49.5) 53 (26.5) 48 (24)	442 (71.2) 91 (14.7) 88 (14.2)	720 (80.4) 88 (9.8) 88 (9.8)	<.001**
CD perianal disease, n (%) Total Isolated perianal disease	70 (35) 2 (1)	170 (27.4) 8 (1.3)	211 (23.5) 20 (2.2)	.003**** .267
Extraintestinal manifestations, n (%)	66 (33)	206 (33.2)	283 (31.6)	.791
Major abdominal surgery, n (%) Resection	117 (58.5)	167 (26.9)	134 (15)	<.001**
Baseline CDAI	330.99 ± 136.92	302.03 ± 110.96	308.07 ± 109.21	.150

Significant *P* values are in bold.

CD, Crohn’s disease; CDAI, Crohn’s disease activity index; GIS, gastrointestinal system.

*Statistically significant differences are between NBE-CEB and NBE-EPB subgroups.

**Statistically significant differences are between NBE-CEB, NBE-EPB, and EPB-CEB subgroups.

***Statistically significant differences are between NBE-CEB and EPB-CEB subgroups.

****Statistically significant differences are between NBE-CEB subgroups.

^a^Results are expressed as mean ± SD, median (interquartile range), or frequency (%).

**Table 4.** The Trend of Patients Diagnosed with Inflammatory Bowel Disease by Periodsa

	Total UC + CD (n = 4224)	UC (n = 2507, 59.4%)	CD (n = 1717, 40.6%)	UC/CD (Total Rate 1.46)	*P*
Era, n (%) 1993-2007 2008-2015 2016-2024	605 (14.3) 1550 (36.7) 2069 (49)	405 (16.2) 929 (37.1) 1173 (46.8)	200 (11.6) 621 (36.2) 896 (52.2)	2.03 1.5 1.31	<.001*

Significant *P* values are in bold.

CD, Crohn’s disease; UC, ulcerative colitis.

*Statistically significant differences are between <1993-2007/2008-2015 and <1993-2007/2016-2024 subgroups.

^a^Results are expressed frequency (%).

**Table 5.** Annual Inflammatory Bowel Disease Diagnosis Frequency in Türkiye Over 3 Decades^a^

	Total IBD (n = 4224)	UC (n = 2507, 59.4%)	CD (n = 1717,40.6%)
Years, n (%)			
<1993	58 (1.4)	42 (1.7)	16 (0.9)
1994	14 (0.3)	11 (0.4)	3 (0.2)
1995	10 (0.2)	6 (0.2)	4 (0.2)
1996	22 (0.5)	13 (0.5)	9 (0.5)
1997	20 (0.5)	12 (0.5)	8 (0.5)
1998	20 (0.5)	11 (0.4)	9 (0.5)
1999	25 (0.6)	16 (0.6)	9 (0.5)
2000	46 (1.1)	32 (1.3)	14 (0.8)
2001	35 (0.8)	30 (1.2)	5 (0.3)
2002	52 (1.2)	29 (1.2)	23 (1.3)
2003	47 (1.1)	27 (1.1)	20 (1.2)
2004	53 (1.3)	37 (1.5)	16 (0.9)
2005	65 (1.5)	45 (1.8)	20 (1.2)
2006	52 (1.2)	36 (1.4)	16 (0.9)
2007	86 (2)	58 (2.3)	28 (1.6)
2008	96 (2.3)	56 (2.2)	40 (2.3)
2009	141 (3.3)	80 (3.2)	61 (3.6)
2010	158 (3.7)	98 (3.9)	60 (3.5)
2011	195 (4.6)	119 (4.7)	76 (4.4)
2012	236 (5.6)	140 (5.6)	96 (5.6)
2013	234 (5.5)	149 (5.9)	85 (5)
2014	249 (5.9)	142 (5.7)	107 (6.2)
2015	241 (5.7)	145 (5.8)	96 (5.6)
2016	234 (5.5)	141 (5.6)	93 (5.4)
2017	227 (5.4)	139 (5.5)	88 (5.1)
2018	275 (6.5)	170 (6.8)	105 (6.1)
2019	332 (7.9)	197 (7.9)	135 (7.9)
2020	236 (5.6)	130 (5.2)	106 (6.2)
2021	304 (7.2)	170 (6.8)	134 (7.8)
2022	218 (5.2)	113 (4.5)	105 (6.1)
2023	165 (3.9)	77 (3.1)	88 (5.1)
2024	78 (1.8)	36 (1.4)	42 (2.4)

CD, Crohn’s disease; IBD, inflammatory bowel disease; UC, ulcerative colitis.

^x^Results are expressed frequency (%).

**Table 6.** Medication Trends According to Periods in Patients with Ulcerative Colitis^a^

	**Non-Biologic Era** (<1993-2007)	**Early Period of Biologics** (2008-2015)	**Common Era of Biologics** (2016-2024)
**Conventional medications, n (%)** Mesalazine oral (n = 1999) Mesalazine enema (n = 1410) Mesalazine suppository (n = 330) Sulfasalazine (n = 108) Budesonide (n = 102) Steroids (n = 768) Thiopurine (n = 941) Methotrexate (n = 35)	297 (14.9) 216 (15.3) 34 (10.3) 33 (30.6) 1 (1) 59 (7.7) 42 (4.5) 1 (2.9)	707 (35.4) 529 (37.5) 127 (38.5) 24 (22.2) 29 (28.4) 221 (28.8) 287 (30.5) 8 (22.9)	995 (49.8) 665 (47.2) 169 (51.2) 51 (47.2) 72 (70.6) 488 (63.5) 612 (65) 26 (74.3)
**Biological agent usage, n (%)** **Total (n = 882)** Adalimumab (n = 313) Infliximab (n = 355) Vedolizumab (n = 116) Ustekinumab (n = 87) Certolizumab (n = 9) Upadacitinib (n = 2)	- - - - - - -	71 (8) 34 (10.9) 37 (10.4) - - - -	811 (92) 279 (89.1) 318 (89.6) 116 (100) 87 (100) 9 (100) 2 (100)

^a^Results are expressed as frequency (%).

**Table 7.** Medication Trends According to Periods in Patients with Crohn’s Disease^a^

	**Non-Biologic Era** (<1993-2007)	**Early Period of Biologics** (2008-2015)	**Common Era of Biologics** (2016-2024)
**Conventional Medications, n (%)** Mesalazine oral (n = 1004) Mesalazine enema (n = 156) Mesalazine suppository (n = 27) Sulfasalazine (n = 148) Budesonide (n = 581) Steroids (n = 664) Thiopurine (n = 1222) Methotrexate (n = 233)	140 (13.9) 21 (13.5) 1 (3.7) 20 (13.5) 20 (3.4) 59 (8.9) 94 (7.7) -	421 (41.9) 60 (38.5) 10 (37) 55 (37.2) 204 (35.1) 232 (34.9) 438 (35.8) 37 (15.9)	443 (44.1) 75 (48.1) 16 (59.3) 73 (49.3) 357 (61.4) 373 (56.2) 690 (56.5) 196 (84.1)
**Biological Agent Usage, n (%)** **Total (n = 1620)** Adalimumab (n = 594) Infliximab (n = 634) Vedolizumab (n = 148) Ustekinumab (n = 178) Certolizumab (n = 64) Upadacitinib (n = 2)	2 (0.1) - 2 (0.3) - - - -	254 (15.7) 163 (27.4) 89 (14) - - 2 (3.1) -	1364 (84.2) 431 (72.6) 543 (85.6) 148 (100) 178 (100) 62 (96.9) 2 (100)

^a^Results are expressed as frequency (%).

## Data Availability

The data underlying this article will be shared on reasonable request to the corresponding author.
